# Society Meetings

**Published:** 1904-08

**Authors:** 


### SOCIETY MEETINGS.

The Association of Military Surgeons will hold its thirteenth annual meeting at Saint Louis, October 10-15, 1904, under the following administration: president, Medical Director John C. Wise, U. S. N., Warrenton, Va.; first vice-president, Surg. Gen. Walter Wyman, P. H. & M. H. S., Washington, D. C.; second vice-president, Major Albert H. Briggs, N. G. N. Y., Buffalo,

N. Y.; third vice-president, Brig. Gen. Robert M. O'Reilly, U. S. A., Washington, D. C.; secretary, Major James Evelyn Pilcher, U. S. V., Carlisle, Pa.; treasurer, Major Herbert A. Arnold, N. G. Pa., Ardmore, Pa.

---

The American Microscopical Society will hold its twenty-fifth annual meeting at Buffalo, August 23, 24 and 25, 1904. The following-named chairman of committees are in charge of details: executive committee, Hon. T. Guilford Smith; committee of arrangements, Dr. William C. Krauss; committee on exhibits, Dr. George E. Fell; committee on finance, Dr. Lee H. Smith.

PROGRAM.

Tuesday, August 23, morning session.—At the Society of Natural Sciences: address of welcome on behalf of the city, Hon. Erastus C. Knight, mayor; on behalf of the Society of Natural Sciences, Hon. T. Guilford Smith, president; on behalf of the medical profession, Dr. Henry R. Hopkins.

Opening of the session, Prof. T. J. Burrill, president of the American Microscopical Society; noon, luncheon; afternoon, scientific meeting; evening, reception.

Wednesday, August 24, morning.—At the Historical Society building: scientific meeting; noon, luncheon at the Park Club; afternoon, scientific meeting; evening, at the Society of Natural Sciences, president's address, microscopical exhibition.

Thursday, August 25, morning.—At the Society of Natural Sciences: final business meeting, election of officers; noon, luncheon; afternoon, trip to Niagara Falls, inspection of power plant.

Mrs. John Miller Horton, chairman of the women's committee, has arranged several trips for the visiting women, including a boat ride on the river, with tea at Falconwood Club, an automobile ride around the city, luncheons, a large reception, and the like.

---

The Medical Society of the County of Chautauqua held its annual meeting at Bemus Point, Wednesday, July 13, 1904, under the presidency of Dr. W. J. French, of Hamlet. No scientific session was held and only such business transacted as was necessary, the day being given over to recreation. A dinner was served at Pickard's hotel, many ladies participating in the entertainments of the day.

---

The American Neurological Association will hold its next annual meeting at Saint Louis, September 15, 16 and 17, 1904, under the presidency of Dr. Frank R. Fry, of Saint Louis. This meeting will be immediately followed by the sessions of the various departments of the congress of arts and sciences, beginning September 17.

---

THE Buffalo Academy of Medicine held meetings during the months of May and June as follows:

*Section on Obstetrics and Gynecology.*—Tuesday evening, May 24. Program: Pancreatitis and cholelithiasis, Stephen Y. Howell. Annual election of officers of this section for ensuing year.

*Section on Ophthalmology.*—Tuesday evening, May 31. Program: The nasopharynx as a portal of entry for germs—The treatment of cancer of the larynx, Roswell Park; discussion lead by F. W. Hinkel. Report of committee on memorial to Dr. Henry D. Ingraham. Election of officers of this section for ensuing year. A collation was served at the close of the meeting.

*Annual Meeting.*—Tuesday evening, June 14. Program: president's address, Our academy—Postpartum hemorrhage, Joseph W. Grosvenor. Official reports for the past year. Election and installation of officers. A collation was served at the close of the meeting.

---

THE Pan-American Medical Congress meets every three years. It was started by Dr. William Pepper, of Philadelphia; Dr. Charles A. L. Reed, of Cincinnati; Dr. Albert Vander Veer, of Albany, and Dr. H. L. E. Johnson, of Washington. The first meeting was held at Washington, September, 1893; the second in Mexico in 1896. The third was to have been held in Venezuela in 1899, but was given up on account of the war in that country. The place of meeting was changed to Cuba, but had to be postponed until 1901 on account of the fever there. These meetings have always been well attended and it is thought that Panama will be an interesting place for the next congress. Further particulars will be sent out from time to time to the journals, together with notifications from the different officers appointed to represent this and other countries.

---

THE American Electrotherapeutic Association will hold its next annual meeting at Saint Louis, September 13-15, 1904, under the presidency of Dr. A. D. Rockwell, of New York. Physicians are invited to apply for membership. Address the secretary, Dr. Clarence E. Skinner, New Haven.

---

THE Lake Keuka Medical and Surgical Association held its fifth annual meeting July 28 and 29, 1904, at Grove Springs, under the presidency of Dr. A. L. Beahan, of Canandaigua. Physicians from Buffalo announced on the program were William C. Krauss, A. A. Hubbell, Roswell Park, Charles E. Congdon, and Floyd S. Crego. Joseph Price, of Philadelphia, was put down to read on Surgery of the tubes and ovaries, and Robert T. Morris, of New York, was listed for a paper entitled, The idea of gross cleanliness in surgery and its harmful results.

---

THE Mississippi Valley Medical Association will hold its thirtieth annual meeting at Cincinnati, October 11, 12, 13, 1904, under the presidency of Dr. Hugh T. Patrick, of Chicago. The headquarters and meeting places will be at the Grand Hotel. The annual orations will be delivered by Dr. William J. Mayo, of Rochester, Minn., in surgery, and Dr. C. Travis Drennen, of Hot Springs, Ark., in medicine. Request for places upon the program, or information in regard to the meeting, can be had by addressing the secretary, Dr. Henry Enos Tuley, Louisville, Ky., or the assistant secretary, Dr. S. C. Stanton, Masonic Temple, Chicago. The usual railroad rates will be in effect.

---

THE Lake Erie Medical Society held its annual meeting at Dunkirk, July 1, 1904, under the presidency of Dr. S. S. Bedient, of Little Valley. The other officers were Dr. A. L. Borden, of Gowanda, vice-president, and Dr. B. E. Smith, of Angola, secretary-treasurer. The following program was observed:

Hydrocephalus, W. C. Krauss, Buffalo; Graves's disease, Edward Torrey, Allegany; Tetanus, with report of two cases, following vaccination, E. M. Coss, Cattaraugus; Clean milk—"A talk,"—N. G. Richmond, Fredonia.

---

THE American Medical Association will hold its next annual meeting at Portland, Ore., July 11-14, 1905. This date has been decided upon, according to the journal of the association, after considerable correspondence, and is so fixed upon as best fitting the vacation season of a majority who may desire to attend. Besides, in July the climate of Portland is all that can be desired, and the scenery is at its best.

